# Bacterial Purification of Sewage

**Published:** 1902-10

**Authors:** 


### Bacterial Purification of Sewage.

B. H. Buxton, *Philadelphia Medical Journal* of April 5th, discusses this important subject in detail, explaining the action of the four most successful systems.

The Cameron system, however, the one chiefly used in England, gives the best results, though even under this system water is not strictly potable in those streams into which the effluent has been allowed to flow. In the above system, the sewage, of which one-tenth is organic matter, is first passed into a large tank (septic tank) in which anerobic bacteria are found. After these have transformed the insoluble proteids into soluble peptones, the liquid is run through beds of charcoal (contact beds) in which aerobic and facultive aerobic bacteria carry the process through its several stages to completion in the form of nitrates, the carbohydrates being changed to water and $CO_2$. The effluent is frequently submitted to the incubation test and those beds not working properly thrown out for a week or two, thus allowing the aerobic bacteria to "strengthen." C. R. M.

